# The impact of the initial operation of PTC in children on recurrence: 9-year experience in a single center

**DOI:** 10.1186/s12957-022-02855-0

**Published:** 2022-12-12

**Authors:** Shaohao Cheng, Ruochuan Cheng, Shunshun Zhao, Min Zhang, Chang Diao, Yunhai Ma, Jun Qian, Yanjun Su

**Affiliations:** Department of Thyroid Surgery, The First Affiliated Hospital of Kunming Medical University, Kunming, 650032 China

**Keywords:** Thyroid carcinoma, Papillary thyroid cancer, Surgery, Treatment, Children

## Abstract

**Purpose:**

To summarize the treatment experience of single-center children with PTC and to explore the influence of initial surgery on the recurrence/metastasis of papillary thyroid carcinoma (PTC) in children.

**Methods:**

A retrospective analysis of PTC case data of children (≤ 18 years old) who were admitted to and received surgical treatment in the First Affiliated Hospital of Kunming Medical University from January 2012 to December 2020.

**Results:**

A total of 64 children with PTC were included, including 45 cases (70.31%) with a single lesion, and 19 cases (29.69%) with multiple lesions (≥ 2 lesions). Fifteen patients relapsed. Univariate analysis found that gender, thyroidectomy scope, central lymph node dissection, and lateral lymph node dissection were risk factors affecting reoperation; multi-factor analysis showed that central lymph node dissection was an independent risk factor affecting reoperation. According to Kaplan–Meier analysis, central lymph node dissection, total thyroidectomy (TT), lobectomy (LT), and disease-free survival (DFS) were statistically significant (*p* = 0.000, *p* = 0.000).

**Conclusion:**

At the time of diagnosis of PTC in children, the rate of lymph node metastasis in the central and lateral cervical regions is high. The vast majority of children with PTC should be treated with TT, and LT is chosen for a small number of patients. CND should be routinely lined.

## Introduction

Differentiated thyroid cancer (DTC) is rare in children, accounting for only 1.4% of childhood malignancies, but it is still the most common endocrine malignancy in children. In addition, its incidence is gradually increasing [1–3]. Children’s thyroid cancer is mostly DTC, including papillary thyroid carcinoma (PTC) and follicular thyroid carcinoma (FTC). Compared with adult thyroid cancer, children’s PTC is usually multifocal, infiltrating the entire gland extensively. At the time of diagnosis, the tumor is larger, the incidence of extrathyroid invasion and lymph node metastasis (LNM) is higher, but the prognosis is good, and there is almost no mortality related to the disease [4, 5].

Due to the characteristics of high invasiveness and good prognosis, surgery is the main treatment for children with PTC. The scope of surgery ranges from lobectomy to total thyroidectomy. Therefore, in 2015, the American Thyroid Association (ATA) released the first version of the guidelines for the management of thyroid cancer in children, aiming to standardize the DTC management of this age group [6]. The guidelines recommend most children’s PTC TT treatment. However, the incidence of permanent hypoparathyroidism and recurrent laryngeal nerve injury after extensive surgery (total or near total thyroidectomy) is higher [7, 8]. Therefore, the purpose of this retrospective study is to assess the risk factors for recurrence of PTC in children, especially the initial surgery.

## Methods

### Patients

Data was collected from 64 cases who were diagnosed with PTC under the age of 18 from January 2012 to December 2020 at the Department of Thyroid Surgery, the first affiliated Hospital of Kunming Medical University. Patients included in this study needed to meet the following criteria:(1)age ≤ 18 years;(2)pathologically confirmed papillary thyroid carcinoma;(3)complete pathological data available.

### Preoperative assessment

All children underwent high-resolution ultrasonography of the thyroid and cervical lymph nodes and Fine needle aspiration biopsy (FNAB) before surgery. Patients with large primary tumors or extensive lymph node metastases underwent neck-enhanced CT scans. The scope of surgery includes thyroidectomy and lymph node dissection. The operation method depends on the preoperative evaluation, intraoperative frozen pathological examination, the judgment of the intraoperative physician, and sometimes the wishes of the guardian. TT was performed for children with primary tumors, lymph node metastasis, or distant metastasis with bilateral, multifocal, T3, or T4 stage lesions. For single-focal T1 and T2 PTC with no clinical evidence of lymph node metastasis (cN0), lobectomy, and isthmus resection were performed.

### Surgical treatment

In our center, children with PTC routinely perform central neck dissection (Central neck dissection, CND), bilateral CND is performed for patients undergoing TT, but ipsilateral CND is performed for patients undergoing lobectomy and isthmus resection. According to the guidelines issued by ATA in 2009: the scope of CND is anterior trachea, bilateral paratracheal, prelaryngeal lymph nodes, etc.; unilateral CND includes prelaryngeal lymph nodes, pretracheal lymph nodes, and ipsilateral paratracheal lymph nodes [9]. Therapeutic lateral neck dissection (LND) was performed in patients with clinically apparent lateral nodal disease, which was defined as cN1b (+). cN1b (+) was diagnosed by preoperative physical examination, US, FNAB, and intraoperative inspection, whereas no clinically apparent lateral nodal disease was defined as cN1b (−).

### Postoperative management and follow-up

All PTC patients received individualized THS suppression therapy after surgery [6], and postoperative RAI treatment was performed on children with T4, N1b, or M1 stages. Patients will be reviewed every 3–6 months. During the follow-up period, patients will undergo thyroid function, neck ultrasound and lung computed tomography (CT). For suspicious lymph nodes or masses in the neck, FNAB is performed. Patients receiving TT and RAI treatments also received thyroid autoantibody tests (TgAb, TPOAb) and the determination of TSH, Tg, and PTH during the follow-up period, and were additionally monitored by US and Rx-WBS to record the patient’s tumor recurrence and clinical status.

### Statistical analysis

All statistical analyses were performed with the SPSS software package (SPSS version 17.0). Continuous variables are represented by mean and standard deviation. The prognostic significance of various factors for remission was assessed using chi-square tests. Student’s *t* test, chi-square test, or Fisher’s exact test, if necessary, was used to compare the two groups. Cox proportional hazard regression models were used to estimate the 95% CI for incident recurrence. *p* < 0.05 were considered statistically significant.

## Results

In this study, a total of 66 children with thyroid cancer were admitted from January 2012 to December 2020, including 64 cases of PTC, 1 case of FTC, and 1 case of MTC. After excluding FTC and MTC cases, a total of 64 children (≤ 18 years old) with PTC were included in this study. The demographics, clinical characteristics, and pathological characteristics of the 64 patients included in this study are summarized in Tables 1 and 2. There were 12 males (18.75%) and 52 females (81.25%). The average age was 14.89 ± 2.99 years (range 6). – 18 years old). Forty-five cases of single lesion (70.31%), 19 cases (29.69%) of multiple lesions (≥ 2 lesions), 48 cases of unilateral cancer (75.00%), 16 cases of bilateral cancer (25.00%), and 27 cases of extrathyroidal invasion (42.19%). According to the 8th edition of AJCC tumor TNM staging, the proportions of T1, T2, T3, and T4 were 40.63%, 17.19%, 26.56%, and 15.63%, respectively; the proportions of N0, N1a, and N1b were 17.19%, 82.81%, and 64.06%, respectively. Six patients (11.11%) had lung metastases at the time of diagnosis (Tables 1 and 2).

**Table 1:** Demographics and clinical characteristics of the patients

Characteristics	No. of patients	%
No. of patients	64	
Gender		
Male	12	18.75%
Female	52	81.25%
Age at diagnosis (years)		
Mean ± SD	14.89 ± 2.99	
Course of the disease		
Median	2 months	
Range	4 days–10 years	
Identification of disease		
Patient/family found	37	57.81%
Physician found	14	21.88%
Abnormal labs/imaging	13	20.31%

**Table 2:** Clinical pathological characteristics of the patients

Characteristics	No. of patients	%
Pathological type		
Classical PTC	60	89.55%
Diffuse sclerosing carcinoma	3	4.69%
Insular carcinoma	1	1.56%
Combined pathology		
Hashimoto’s thyroiditis, HT	20	31.25%
Nodular Goiter	11	17.19%
Multifocality		
Single	45	70.31%
Multiple(≥ 2)	19	29.69%
Tumor location		
Unilateral	48	75.00%
Left lobe	29	45.31%
Right lobe	19	29.69%
Bilateral	16	25.00%
Tumor size		
≤ 1 cm	15	23.44%
> 1, ≤ 2 cm	20	31.25%
> 2, ≤ 4 cm	26	40.63%
> 4 cm	3	4.69%
Extrathyroidal invasion		
Yes	27	42.19%
No	37	57.81%
T classification		
T1	26	40.63%
T1a	10	15.63%
T1b	16	25.00%
T2	11	17.19%
T3	17	26.56%
T3a	1	1.56%
T3b	16	25.00%
T4	10	15.63%
T4a	10	15.63%
T4b	0	0%
N classification (imaging and clinical) ^a^		
N0	11	17.19%
N1a	53	82.81%
N1b	41	64.06%
M classification (imaging and clinical)^b^		
M0	58	84.38%
M1	6	11.11%
RAI after surgery		
Yes	30	46.88%
No	34	53.13%

^a^For N classification, N0 no any evidence of regional lymph node metastasis; N1a and N1b were proved pathologically

^b^For M classification, M0 no any evidence of distant metastasis; M1 was confirmed by Rx-WBS

### Lymph node metastasis in different surgical approaches

We routinely performed CND on 64 patients admitted and performed bilateral CND on patients undergoing TT, but performed ipsilateral CND on patients undergoing lobectomy and isthmus resection, and performed therapeutic LND on patients with cN1b (+). The dissected cervical lymph nodes will be sent for pathological examination. We found that the LNM rate in the TT group was significantly higher than that in the LT group (91.11% vs. 21.05%, *χ*2 = 31.412, *p* = 0.000). At the same time, the entire group of data showed that the central lymph node metastasis (CLNM) rate was also significantly higher than the lateral lymph node metastasis (LLNM) rate (70.31% vs. 53.13%, *χ*2 = 4.001, *p* = 0.045) (Table 3).

**Table 3:** Lymph node metastasis rate in different surgical approaches

Surgical approach	*N*	CLNM [*n*/%]	LLNM [*n*/%]	LNM [*n*/%]
Total thyroidectomy	45	41/91.11%	34/75.56%	41/91.11%
Lobectomy	19	4/21.05%	–	4/21.05%^a^
L-Lobectomy	10	1/10%	–	1/10%
R-Lobectomy	9	3/33.33%	–	3/33.33%
Total	64	45/70.31%	34/53.13%^b^	45/70.31%

^a^Compared with LNM rate of TT, *χ2* = 31.412, *p* = 0.000

^b^Compared with CLNM rate, *χ2* = 4.001, *p* = 0.045

### Lymph node metastasis based on clinical N1b stage

We performed preoperative N staging on 64 patients, cN1b (−): 23 people, cN1b (+): 41 people. CND was routinely performed in cN1b (−) patients, unilateral CND was performed in 10 patients, and bilateral CND was performed in 13 patients. Among them, 50% of patients with unilateral CND had ipsilateral CLNM, while the rates of ipsilateral and contralateral CLNM in patients with bilateral CND were 61.54% and 46.15%, respectively. In addition to routine CND, patients with cN1b (+) were also treated with therapeutic LND. It was found that the rates of CLNM and LLNM in patients with cN1b (+) were higher on the ipsilateral side than on the contralateral side (see Table 4). In addition, we also found that the LNM rate of cN1b (−) was significantly lower than that of cN1b (+) (56.52% vs 95.12%, *χ*2 = 14.411, *p* = 0.000). At the same time, the rates of ipsilateral CLNM and LLNM of the entire group of patients were significantly higher than the contralateral central area (81.25% vs 60.94%, *χ*2 = 6.425, *p* = 0.011) and the contralateral cervical area (60.94% vs 26.56%, *χ*2 = 15.365, *p* = 0.011).

**Table 4:** Relationship between clinical N1b stage, CND, and LND

Clinical N1b stage	Neck dissection	*N*	CLNM [*n*/%]		LLNM [*n*/%]		
			Ipsilateral^a^	Contralateral	Ipsilateral^a^	Contralateral	LNM [*n*/%]
cN1b (−)	CND	23	13/56.52%	6/26.09%	–	–	13/56.52%
	Unilateral CND	10	5/50.00%	–	–	–	
	Bilateral CND	13	8/61.54%	6/46.15%	–	–	
cN1b (+)	LND	41	39/95.12%	33/80.49%	39/95.12%	17/85.00%	39/95.12%^b^
	Unilateral LND	21	20/95.24%	16/76.19%	20/95.24%	–	
	Bilateral LND	20	19/95.00%	17/85.00%	19/95.00%	17/85.00%	
Total		64	52/81.25%^c^	39/60.94%	39/60.94%^d^	17/26.56%	

^a^In patients with bilateral lesions, the larger side of the tumor is regarded as ipsilateral and the smaller side of the lesion as contralateral

^b^vs LNM, *χ2* = 14.411, *p* = 0.000

^c^vs Contralateral CLNM，*χ2* = 6.425, *p* = 0.011

^d^vs Contralateral LLNM，*χ2* = 15.365, *p* = 0.011

### Risk factors of reoperation

**Fig. 1:**
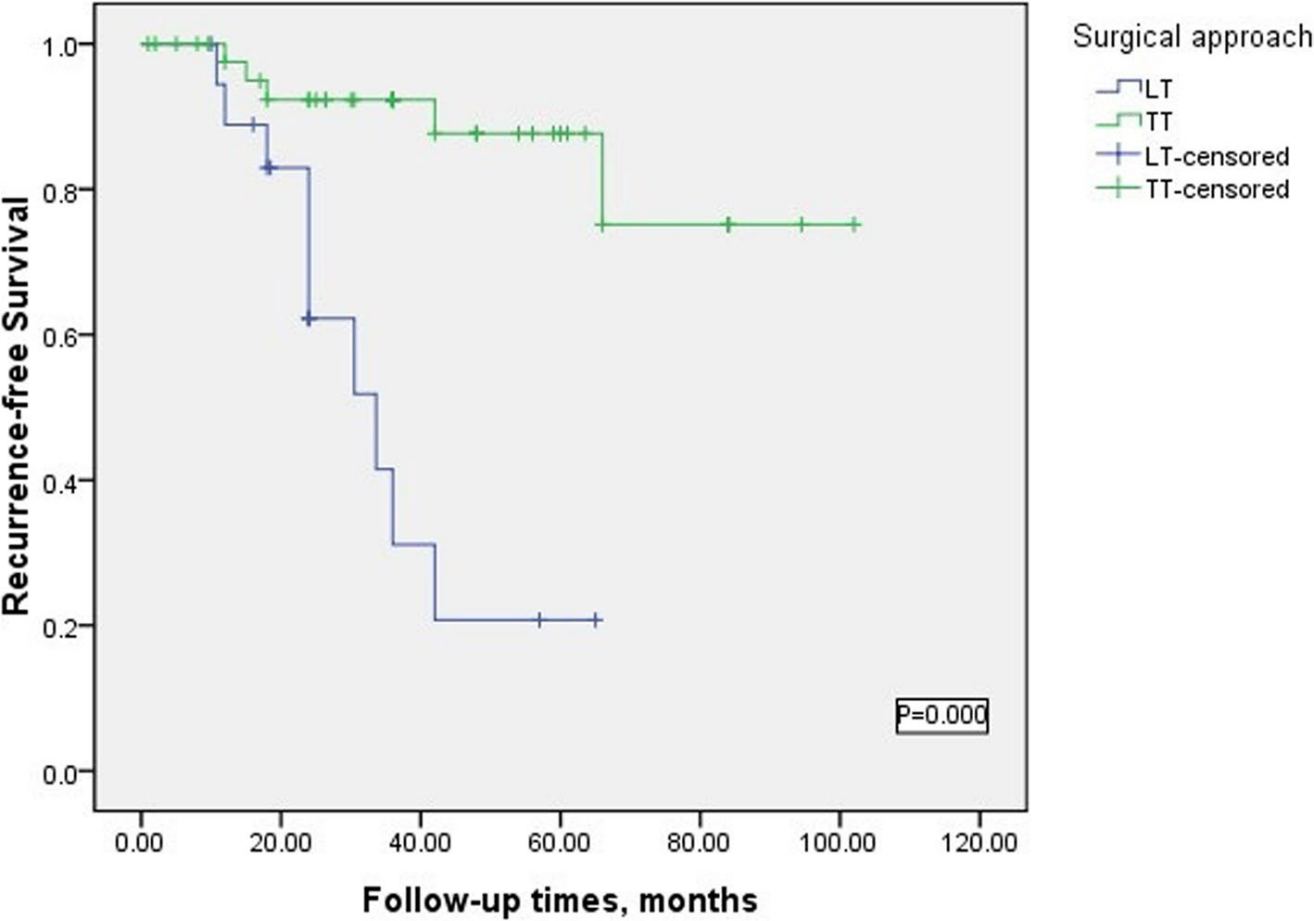
Kaplan–Meier survival curve analysis of the surgery approach on disease recurrence-free survival

**Fig. 2:**
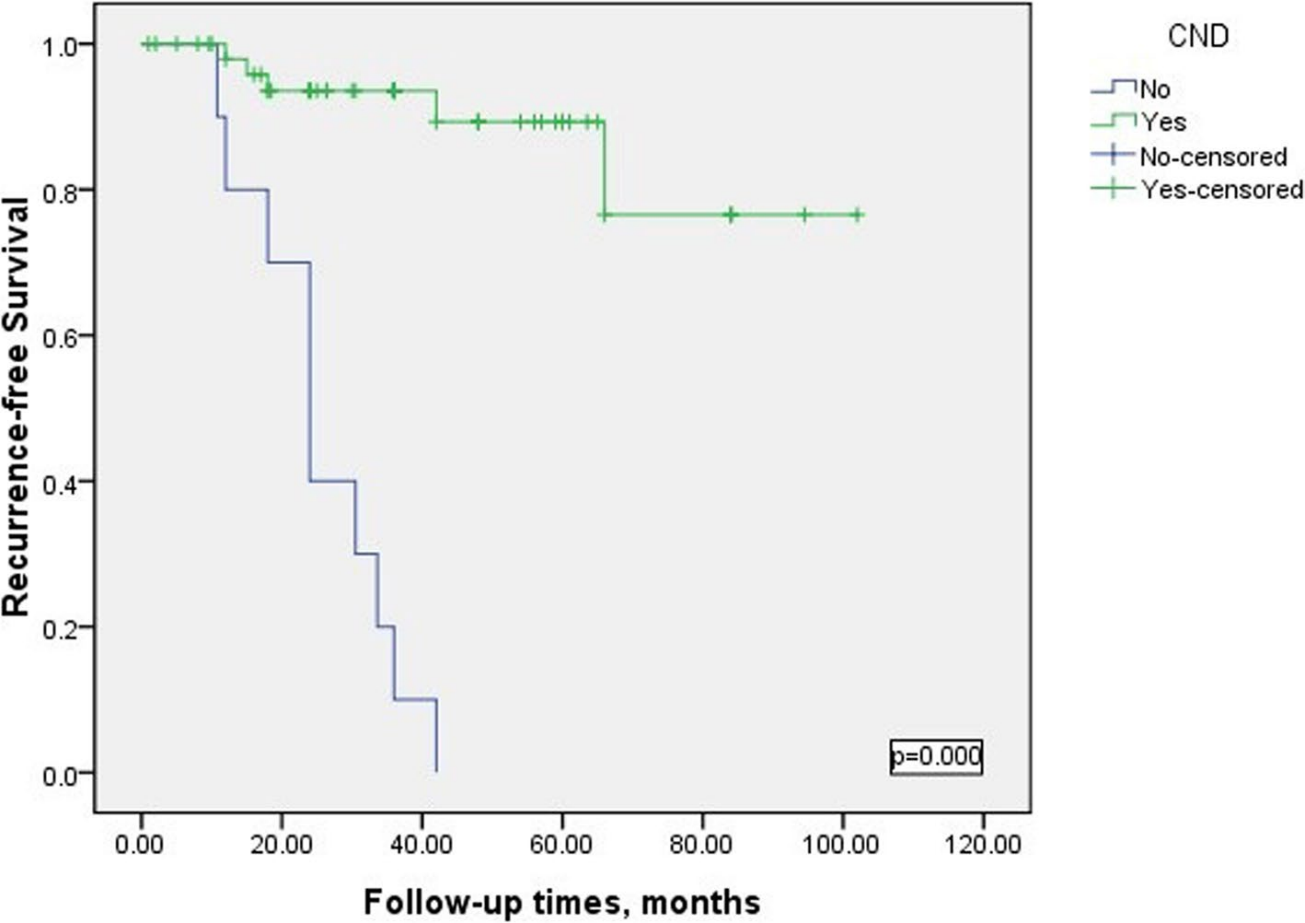
Kaplan–Meier survival curve analysis of the CND on disease recurrence-free survival

The median follow-up time of our cohort was 36.52 months. During the follow-up period, no permanent hypoparathyroidism occurred, and no deaths occurred. In addition, TgAb, TPOAb, TSH, Tg, and PTH were also tested, and US and Rx-WBS were additionally performed to monitor tumor recurrence. We took tumor recurrence and metastasis as the endpoint, and found that 15 children had persistent or recurrent disease. The frozen pathological detection of residual cancer after reoperation was 10 cases (66.67%) were children who received LT (all with residual thyroid recurrence, DFS was 48m, 8m, 84m, 72m, 72m, 60m, 24m, 84m, 60m, and 48m), 5 cases were children who received TT (cluding 3 recurrences in the central area, 5 recurrences in the cervical lateral area, and 1 recurrence in the residual thyroid gland, including overlapping cases, DFS is 60m, 24m, 60m, 36m, and 48m). Univariate Cox analysis found that gender, extent of thyroidectomy, central lymph node dissection, and lateral lymph node dissection were risk factors affecting reoperation; after multi-factor Cox analysis, central lymph node dissection was an independent risk factor affecting reoperation (Table 5). In the Kaplan–Meier survival curve of PTC patients, BTT and CND are related to DFS (*p* = 0.000, *p* = 0.000) (Figs. 1 and 2).

**Table 5:** Univariable and multivariable Cox analysis of factors associated with reoperation

Variables	Univariate		Multivariate	
	95% CI	*P* value	95% CI	*P* value
Gender (M/F)	0.130 (0.033–0.513)	0.002		
Age (0–12, 13–18)	0.457 (0.139–1.501)	0.192		
Thyroidectomy (TT/LT)	0.113 (0.035–0.369)	0.000		
cN1b (yes/no)	2.759 (0.689–11.048)	0.142		
RAI (yes/no)	0.155 (0.035–0.691)	0.014		
CND (yes/no)	0.05 (0.015–0.167)	0.000	0.05 (0.015–0.167)	0.000
LND (yes/no)	0.297 (0.092–0.957)	0.042

## Discussion

Differentiated thyroid cancer is the most common endocrine tumor in children, accounting for about 1.4% of malignant tumors in children. According to SEER, the incidence of thyroid cancer in children and adolescents rose from 0.48/100,000 in 1973 to 1.14/100,000 in 2013. The incidence increased by 1.1% per year before 2006. In 2013, it increased by 9.6% per year [1], and its incidence has been the same as that of adults, showing an equivalent upward trend [5], which may reflect the improvement of imaging diagnostic technology, the excessive use of imaging and the increase in actual incidence. In addition, environmental and personal factors also affect the incidence [1], making thyroid cancer one of the more common malignant tumors in children and adolescents. Secondly, compared with adults, there are big differences in the biological characteristics, clinical characteristics, and long-term prognosis of thyroid cancer in children. PTC in children and adolescents is mainly multifocal and aggressive, and it is very easy to invade the thyroid capsule, directly involving the recurrent laryngeal nerve, trachea, blood vessel, and esophagus. Based on the above characteristics of children, the current treatment mostly refers to the first edition of the “Guidelines for the Management of Children's Thyroid Nodules and Differentiated Thyroid Cancer” published by ATA in 2015 [6]. The guidelines recommend that based on the high incidence of bilateral and multifocal PTC in children, surgery is considered to be the most important treatment for children with PTC, and TT is the first choice for most children with PTC [6]. Although TT is the main treatment for most children with PTC, the scope of thyroid resection is still controversial. The main problem is the impact of surgical resection on tumor recurrence and potential complications (such as transient/permanent postoperative thyroidectomy). Hypoparathyroidism and recurrent laryngeal nerve injury) [7, 10].

In this study, we found that 70.31% of patients had lymph node metastasis in the preoperative diagnosis, and intraoperative frozen biopsy also verified the idea of a high lymph node metastasis rate in children [11]. According to the preoperative evaluation, 45 patients were treated with TT and 19 patients were treated with LT. In the postoperative follow-up, we found that the recurrence rate of TT treatment was lower than that of LT (33.33% vs. 66.67%, *P* = 0.000), the difference between the two was statistically significant, and none of the children treated with TT had serious or permanent complications. In the TT group, the main cervical lymph node metastasis, in contrast, the LT group, the contralateral glandular lobe, and cervical lateral lymph node metastasis, which may be due to the extent of our lymph node dissection. In addition, in univariate analysis, the scope of thyroidectomy and central lymph node dissection were risk factors for recurrence after surgery (*p* = 0.000, *p* = 0.000), but multivariate analysis found that CND was a significant risk factor for recurrence after surgery (95% CI, 0.015–0.167, *p* = 0.000), and the scope of surgical resection and prognosis were not statistically significant, which may be due to the differences in patient populations in this study. However, the KM survival curve found that TT and LT were statistically significant with DFS (*p* = 0.000). Wang et al. [8] reported that TT could not reduce the recurrence rate, but only increased surgical complications. Spinelli et al. [12] considered single focus DTC in children with no distant metastasis can choose lobectomy. However, Amarasinghe et al. [13] reported that the incidence of residual cancer may increase up to 30% without total thyroidectomy. And, some oncologists advocate bilateral thyroidectomy with the main rea-sons as follows: (1) the bilateral lobes of the thyroid are not separate and are linked to each other into an organ; you must remove the entire organ in order to reduce the cancer relapse; (2) pediatric differentiated thyroid cancer has the characteristics of multifocal lesions. However, in actual clinical work, during initial surgical treatment It is difficult to carry out accurate risk stratification for each patient, and it is also difficult to predict the risk of recurrence during surgery. In this regard, from the long-term recurrence and based on the high lymph node metastasis rate in children, we believe that TT is performed for most children with PTC, and lobectomy can be selected for a small number of patients.

The central area is the most common metastatic site of PTC, and children with PTC are more likely to have CLNM [8, 14, 15], which increases the risk of lung metastasis [16–18]. Children’s guidelines recommend that CND should be performed for children who have had malignant cytology and are found to have obvious extrathyroidal invasion and/or local regional lymph node metastasis during preoperative staging or during surgery; for those without serious extrathyroidal invasion and/or local metastasis Patients should choose preventive ipsilateral or bilateral CND [6]. In this study, we performed CND prophylactically, bilateral CND was performed on patients with TT, and ipsilateral CND was performed on patients with LT. It was found that 50% of patients with unilateral CND had ipsilateral CLNM, while the rates of ipsilateral and contralateral CLNM in patients with bilateral CND were 61.54% and 46.15%, respectively. In addition, CND can reduce the risk of persistent or locally recurring tumors, thereby prolonging DFS, and may improve the efficacy of RAI [16, 19], and TT + preventive CND can increase 5-year and 10-year DFS to 95% [20]. Based on children's high CLNM, we recommend routine CND for children with PTC. Children who receive TT should be given bilateral CND under the premise of controllable surgical complications, and children who undergo lobectomy should also be given ipsilateral preventive CND.

## Conclusions

This study shows that a comprehensive preoperative assessment of the primary tumor and lymph node metastasis should be carried out before the treatment of PTC in children to optimize the surgical plan. TT should be chosen for the vast majority of children with PTC, and LT should be chosen for a small number of patients. Children’s PTC should be routinely performed CND. Bilateral CND is the first choice. Preventive unilateral CND is feasible for children with high surgical risk or only LT.

## Data Availability

The study data of validation cohort used and/or analyzed during the current study are available from the First Affiliated Hospital of Kunming Medical University, China.
